# Casticin inhibits breast cancer cell migration and invasion by down-regulation of PI3K/Akt signaling pathway

**DOI:** 10.1042/BSR20180738

**Published:** 2018-11-30

**Authors:** Li Fan, Yi Zhang, Qiuhong Zhou, Ying Liu, Baolan Gong, Jieyu Lü, Hui Zhu, Guijuan Zhu, Yingping Xu, Guangrong Huang

**Affiliations:** 1 Department of Obstetrics and Gynecology, Renmin Hospital, Hubei University of Medicine, Shiyan 442000, Hubei Province, China; 2 Department of Obstetrics and Gynecology, Zhuxi Maternal and Child Health Hospital, Shiyan 442000, Hubei Province, China; 3 Department of Gynecology, Shenzhen Bao’an Traditional Chinese Medicine Hospital Group, Shenzhen 518133, Guangdong Province, China

**Keywords:** Breast cancer, Casticin, invasion, MMP-9, PI3K/Akt

## Abstract

Casticin is one of the major active components isolated from *Fructus viticis*. Increasing studies have revealed that casticin has potential anticancer activity in various cancer cells, but its effects on breast cancer cell migration and invasion are still not well known. Therefore, the ability of cell migration and invasion in the breast cancer MDA-MB-231 and 4T1 cells treated by casticin was investigated. The results indicated that casticin significantly inhibited cell migration and invasion in the cells exposed to 0.25 and 0.50 µM of casticin for 24 h. Casticin treatment reduced matrix metalloproteinase (MMP) 9 (MMP-9) activity and down-regulated *MMP-9* mRNA and protein expression, but not MMP-2. Casticin treatment suppressed the nuclear translocation of transcription factors c-Jun and c-Fos, but not nuclear factor-κB (NF-κB), and decreased the phosphorylated level of Akt (p-Akt). Additionally, the transfection of Akt overexpression vector to MDA-MB-231 and 4T1 cells could up-regulate MMP-9 expression concomitantly with a marked increase in cell invasion, but casticin treatment reduced Akt, p-Akt, and MMP-9 protein levels and inhibited the ability of cell invasion in breast cancer cells. Additionally, casticin attenuated lung metastasis of mouse 4T1 breast cancer cells in the mice and down-regulated MMP-9 expression in the lung tissues of mice treated by casticin. These findings suggest that MMP-9 expression suppression by casticin may act through inhibition of the phosphatidylinositol 3-kinase (PI3K)/Akt signaling pathway, which in turn results in the inhibitory effects of casticin on cell migration and invasion in breast cancer cells. Therefore, casticin may have potential for use in the treatment of breast cancer invasion and metastasis.

## Introduction

Breast cancer is the most frequently diagnosed cancer amongst women behind lung cancer [1]. Over the past two decades, although the treatment for breast cancer has substantially improved, its metastasis is still a major cause of mortality and poor prognosis [2,3]. Cell migration and invasion are the key steps for breast cancer metastasis [4]. Thus, suppression of cancer cell migration and invasion represents an important therapeutic target. The development of new therapeutic agents to prevent cancer cell migration and invasion is highly desirable.

**Figure 1:**
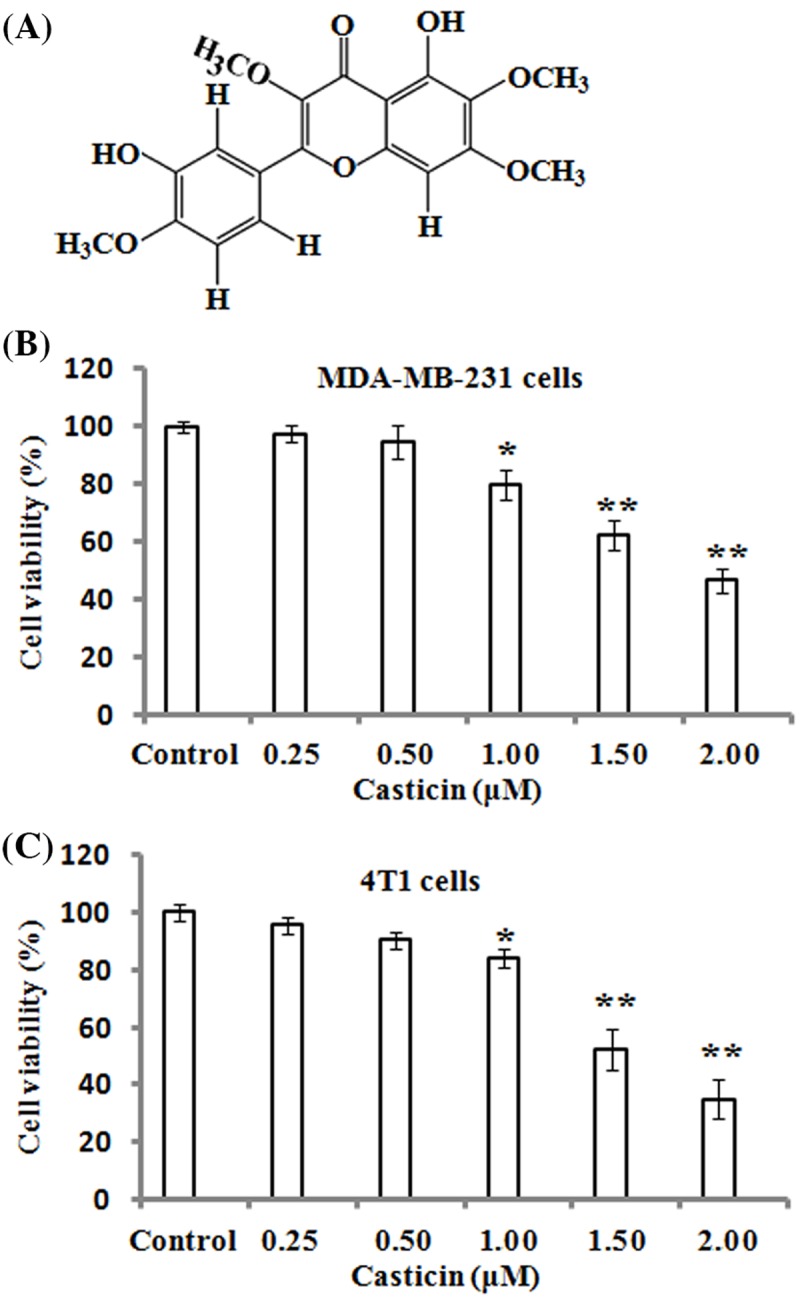
Effects of casticin on the viability of breast cancer cells (**A**) Chemical structure of casticin. (**B**,**C**) MDA-MB-231 and 4T1 cells were respectively treated with various concentrations (0, 0.25, 0.50, 1.00, 1.50, and 2.00 µM) of casticin for 24 h, and cell viability was determined by MTT assays. The results represented the mean ± S.D. of three independent experiments. *P<0.05 and **P<0.01 compared with the control (0 µM of casticin).

Casticin (5,30-dihydroxy-3,6,7,40-tetramethoxyflavone), a polymethoxyflavone, is the main active ingredient of *Fructus Vitcis Simplicifoliae*, which is also used as a folk medicine to be an anti-inflammatory agent and treat certain cancers in China [5]. The chemical structure of casticin is shown in Figure 1A. It has been shown that casticin has extensive anticancer pharmacological activities for various cancers including leukemia [6], ovarian cancer [7], colon cancer [8], lung cancer [5], hepatocellular carcinoma cells [9], and so on [10,11]. The inhibitory effects of casticin on cell proliferation and apoptotic induction in various cancer cells are primarily mediated by the generation of mitochondria-dependent reactive oxygen species (ROS) and activation of caspase-3 and -9 [12]. Additionally, the anti-inflammatory properties of casticin have also been confirmed in several studies through the nuclear factor-κB (NF-κB), Akt, and mitogen-activated protein kinase (MAPK) signaling pathways [13,14].

Recently, it was found that casticin could inhibit the invasion of lung cancer stem-like cells through down-regulation of Akt phosphorylation (p-Akt) and matrix metalloproteinase (MMP) 9 (MMP-9) activity [15]. In addition, casticin was found to suppress the migration and invasion ability of human melanoma cells by down-regulating MMP-2 and NF-κB p65 expression [16]. In mouse melanoma B16F10 cells, casticin treatment also showed that it impaired cell migration and invasion, decreased the expressions of MMP-9, MMP-2, and MMP-1, and inhibited the phosphatidylinositol 3-kinase (PI3K)/Akt and NF-κB signaling pathways [17]. A recent study demonstrated that casticin treatment induced apoptosis in breast cancer cells via the activation of forkhead box O3 (FOXO3a) and the repression of forkhead box protein M1 (FOXM1) [18]. Currently, to the best of our knowledge, no information about casticin-mediated migration and invasion in breast cancer cells is available. Accordingly, in the present study, we investigated the inhibitory effects of casticin on breast cancer cell migration and invasion and explored its related mechanisms. The results demonstrated, for the first time, that casticin significantly suppressed the migration and invasion of breast cancer cells *in vitro* and inhibited breast cancer cell metastases to lung in mice.

## Materials and methods

### Chemicals and reagents

Casticin, MTT, and DMSO were purchased from Sigma–Aldrich (St. Louis, MO, U.S.A.). Casticin was dissolved in DMSO and stored at −20°C. The final content of DMSO was kept at 0.1% in all cell cultures, which did not demonstrate a significant effect on cell proliferation and morphology (data not shown). Dulbecco’s modified Eagle’s medium (DMEM) and Matrigel were obtained from Invitrogen Life Technologies (Carlsbad, CA, U.S.A.) and Collaborative Biomedical Products (Bedford, MA, U.S.A.), respectively. The PI3K inhibitor LY294002 was purchased from Selleck Chemicals (Houston, TX, U.S.A.). The primary antibodies against MMP-2, MMP-9, NF-κB P65, c-Jun, c-Fos, PI3K, Akt, p-Akt, P38, p-P38, c-Jun N-terminal kinase (JNK), p-JNK, extracellular signal-regulated kinase (ERK), p-ERK, β-actin, and Lamin B were purchased from Cell Signal Technology (Beverly, MA, U.S.A.).

### Cell culture

Human breast cancer cell line MDA-MB-231 and mouse breast cancer cell line 4T1 were both obtained from China Center for Type Culture Collection (Wuhan, China), and maintained in DMEM supplemented with 10% FBS, 100 U/ml penicillin, and 100 µg/ml streptomycin (HyClone, UT, U.S.A.). The cells were cultured at 37°C in a humidified incubator with 5% CO_2_ and 95% air.

### Cell viability

Cell viability was assayed by the MTT method. Briefly, MDA-MB-231 and 4T1 cells were respectively seeded in 96-well plates at a density of 1 × 10^4^ cells/well and culture for 12 h, followed by treatment with various casticin concentrations (0, 0.25, 0.50, 1.00, 1.50 and 2.00 µM) for 24 h. The MTT solution (0.1 mg/ml) was then added for another 4 h culture, and the medium was subsequently removed. Next, 200 µl of DMSO was added to dissolve the formed formazan crystals. The absorbance of each well was measured at 570 nm by a microplate reader (Bio-Tek, Norcross, GA, U.S.A.).

### Wound healing assay

MDA-MB-231 and 4T1 cells were grown to a 90% confluent monolayer in six-well culture dishes, and scratched with a P-10 pipette tip to create wounds, followed by incubation with 0, 0.25, and 0.50 µM of casticin for 24 h. Phase contrast images were taken by a microscopy system (Olympus, Japan). The cells that migrated into the denuded zone of each dish were quantitated in a field of view using ImageJ software (NIH, Bethesda, MA, U.S.A.). The experiments were independently performed three times.

### *In vitro* cell invasion assay

Cell invasion was performed by modified Boyden chamber method. Briefly, MDA-MB-231 or 4T1 cells were harvested and resuspended in serum-free DMEM, and 200 µl of cell suspension (5 × 10^5^ cells/ml) containing 0, 0.25, and 0.50 µM of casticin were then seeded into the top chambers with 8-µm pore size polycarbonate membrane filters that were pre-coated with Matrigel (25 mg/ml). Standard DMEM with 10% FBS was added into the bottom chamber. After 24 h incubation, the cells on the upper surface of the membrane were removed with cotton swabs, and the cells that invaded the lower surface of the membrane were fixed with methanol and stained with Hematoxylin and Eosin (H&E) solution. Cell numbers were counted in four randomly selected fields under a light microscope at ×400 magnification.

### Gelatin zymography

The activities of MMP-2/9 in the conditional medium were analyzed with gelatin zymography protease assays. In brief, the cells were incubated with 0, 0.25, and 0.50 µM of casticin in serum-free DMEM for 24 h, followed by the collection of supernatants which were mixed with loading buffer (1% sucrose, 2.5% SDS, and 4 µg/ml Phenol Red) without reduction agents. The samples were then loaded on 8% polyacrylamide gels copolymerized with gelatin (1 mg/ml). After electrophoresis, the gels were washed twice with 2.5% Triton X-100, and then incubated in collagenase buffer (10 mM CaCl_2_, 50 Tris/HCl pH 7.6) for 48 h at 37°C, followed by staining with 0.5% Coomassie Blue for 30 min at room temperature, and destained in 10% methanol and 10% acetic acid until clear bands were revealed. The gelatinolytic activity was then detected as clear bands against the blue background [19].

### Real-time quantitative PCR

The cells were cultured in the absence or presence of 0.25 and 0.50 µM casticin for 24 h, and then collected for total RNA extraction using TRIzol reagent (Life Technologies, Carlsbad, CA, U.S.A.), followed by reverse transcription into cDNA using a PrimeScript RT Master Mix kit (Takara, Tokyo, Japan). Quantitative PCRs were performed with an ABI7900HT machine (Applied Biosystems, France) in a final volume of 15 µl according to the manufacturer’s instructions using SYBR green fluorescence signal detector. The specific primers were listed as follows [20]: MMP-2 forward 5′-TGAGCTCCCGGAAAAGATTG-3′ and reverse 5′-TCAGCAGCCTAGCCAGTCG-3′; MMP-9 forward 5′-TCCCTGGAGACCTGAGAACC-3′, and reverse 5′-CGGCAAGTCTTCCGAGTAGTT-3′; glyceraldehyde-3-phosphate dehydrogenase (GAPDH) forward, 5′-CCATCACCATCTTCCAGGAG-3′ and reverse 5′-CCTGCTTCACCACGTTCTTG-3′. The relative expression levels of *MMP-2/9* mRNA were determined using the *C*_t_ method by normalizing their mRNA *C*_t_ values to those for GAPDH (Δ*C*_t_).

### Western blot analysis

The cells were treated with 0, 0.25, and 0.50 µM of casticin for 24 h or pre-treated with LY294002 (10 µM) for 2 h prior to the addition of casticin, and then the cells were harvested and prepared for cytosolic and nuclear protein extraction using a cytoplasmic and nuclear protein extraction kit (BioTeke Corporation Co., Beijing, China) according to the manufacturer’s instructions. After the protein content was measured using the Bradford method, equal amounts of protein (20 µg) were separated on 10% SDS/polyacrylamide gel for electrophoresis, and transferred on to a polyvinylidene membrane (Millipore, Bedford, MA, U.S.A.). The membrane was blocked in 5% nonfat skim milk, and probed with primary antibodies against MMP-2, MMP-9, NF-κB P65, c-Jun, c-Fos, PI3K, Akt, p-Akt, P38, p-P38, JNK, p-JNK, ERK, p-ERK, β-actin, Lamin B, and specific secondary antibodies. The band signals were detected using an ECL kit (Amersham Biosciences, NJ, U.S.A.) according to the manufacturer’s instructions. The protein levels were quantitated by densitometric analysis using ImageJ software (NIH, Bethesda, MA, U.S.A.).

### Transient transfection

A transient transfection assay was performed as previously described [21]. MDA-MB-231 and 4T1 cells were respectively plated on to six-well plates and grown to 80% confluence. The cells were then transfected with an expression vector pUSE (Amp) for *Akt1* cDNA or its corresponding control empty pUSE vector (Amp) using Lipofectamine 2000 (Invitrogen, Camarillo, U.S.A.) according to the manufacturer’s instructions. After 12 h transfection, the cells were subsequently washed with PBS and replenished in DMEM containing 20% serum, followed by incubation with 0.50 µM of casticin for 24 h, and then expanded for further studies.

### *In vivo* model of hematogenous metastatic dissemination

An animal model of experimental lung metastasis assay was performed. Briefly, 6-week-old female Balb/c mice were obtained from Guangdong Medical Laboratory Animal Center (Guangzhou, Guangdong, China), and housed in a specific pathogen-free facility with sterilized food and water supply. All the procedures with mice were reviewed and approved by the Animal Care and Use Committee of Hubei University of Medicine. The mice were anesthetized with 20 mg/kg xylazine and 100 mg/kg ketamine by intraperitoneal (i.p.) injection, and inoculated in the left ventricle of the heart with 2.0 × 10^5^ 4T1 cells in 100 µl of serum-free DMEM as described previously [22]. The mice (*n*=6) were then treated with casticin (10 mg/kg) through i.p. injection once every 2 days for 4 weeks, and the mice left untreated served as the control group (*n*=6). All mice were killed at 28 days after treatment, and the lung tissues were stained by routine H&E. The sections were photographed and analyzed with ImageScope software (Aperio Technologies, Vista, CA). The number of lung metastases was counted in a blinded fashion.

### Immunohistochemistry

To evaluate the expression of MMP-2 and MMP-9 proteins in the excised lung tissues, immunohistochemistry in paraffin-embedded sections was performed. In brief, the sections (5 µm) were deparaffinized and antigen retrieval was performed in citrate buffer at 98°C for 20 min, followed by incubation in 0.3% methanol/hydrogen peroxide for 15 min to quench endogenous peroxidase. Non-specific proteins were blocked with 2.5% normal horse serum for 20 min. The sections were then incubated with primary antibodies against MMP-2 and MMP-9 overnight at 4°C, followed by incubation with horseradish peroxidase-labeled secondary antibody for 30 min and avidin–biotin–peroxidase complex (Vector Laboratories Ltd., Peterborough, U.K.) for 1 h. Color was then developed by incubation with 3,3-diaminobenzidine solution (Sigma–Aldrich, U.S.A.), followed by counterstaining with Hematoxylin. The specific staining of MMP-2 and MMP-9 in the sections was observed using a microscope with a digital camera.

### Statistical analysis

Data are presented as the mean ± S.D. The comparison of the means of two groups was performed by Student’s *t*test for statistical analysis. A *P*-value <0.05 was considered statistically significant.

## Results

### Effects of casticin on the viability of breast cancer cells

To assess the cytotoxicity of casticin on MDA-MB-231 and 4T1 cells, cell viability was detected by MTT assays after the cells were treated with various concentrations of casticin (0, 0.25, 0.50, 1.00, 1.50, and 2.00 µM) for 24 h. As shown in Figure 1B,C, the results demonstrated that a significant inhibitory effect on the viability of MDA-MB-231 and 4T1 cells was observed when the cells were treated with 1.00–2.00 µM of casticin. However, the treatment with 0.25 and 0.50 µM of casticin did not result in a significant reduction in viability in the MDA-MB-231 and 4T1 cells. Based on these findings, 0.25 and 0.50 µM of casticin were chosen for the following experiments.

### Effects of casticin on the motility and invasion of breast cancer cells

**Figure 2:**
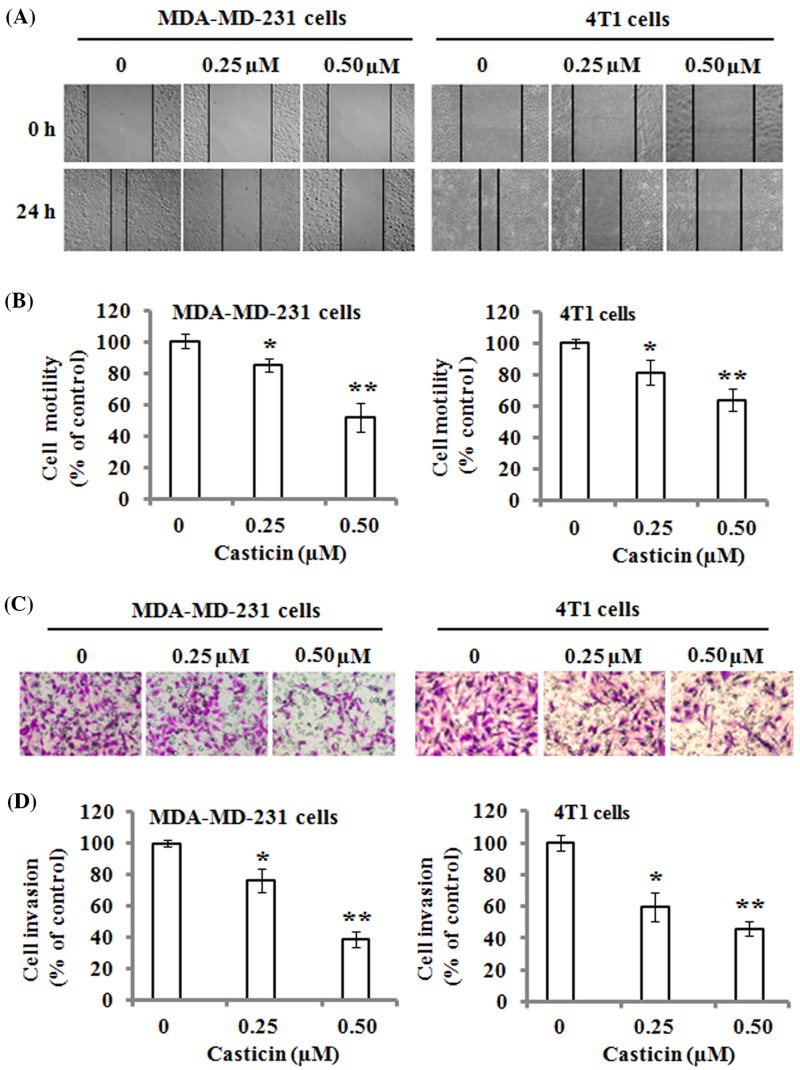
Effects of casticin on cell migration and invasion The monolayers of MDA-MB-231 and 4T1 cells were respectively scratched with a pipette tip, and incubated with 0, 0.25, and 0.50 µM of casticin for 24 h. (**A**) Representative images of wound healing. Original magnification was ×100. (**B**) The number of cells migrated to the denuded zone was quantitated, and normalized to that of the control. (**C**) Cell invasion was analyzed with a Matrigel-coated Boyden chamber. MDA-MB-231 and 4T1 cells were respectively treated with 0, 0.25, and 0.50 µM of casticin for 24 h, and then analyzed as described in the ‘Materials and methods’ section. Representative photomicrographs of the membrane-associated cells were assayed by H&E staining. Original magnification was ×400. (**D**) Cell invasion ability was quantitated. Data represent the mean ± S.D. of three independent experiments. *P<0.05 and **P<0.01 compared with the control.

The effects of casticin on the migration of MDA-MB-231 and 4T1 cells were analyzed by wound-healing assays. The results demonstrated that there were fewer cells in the wounded area as compared with the control cells when the cells were exposed to 0.25 and 0.50 µM of casticin for 24 h (Figure 2A). The quantitative data revealed that casticin significantly suppressed the motility ability of MDA-MB-231 and 4T1 cells (Figure 2B). On the other hand, transwell Matrigel invasion assays showed that casticin strongly inhibited cell invasion in the MDA-MB-231 and 4T1 cells in the Boyden chamber (Figure 2C). The quantitative results indicated that the invasive abilities of MDA-MB-231 and 4T1 were significantly reduced by casticin treatment compared with the control cells (Figure 2D). These data indicate that casticin has a strong suppressive ability on the migration and invasion of breast cancer cells.

### Effects of casticin on the activities and expression of MMP-2/9

**Figure 3:**
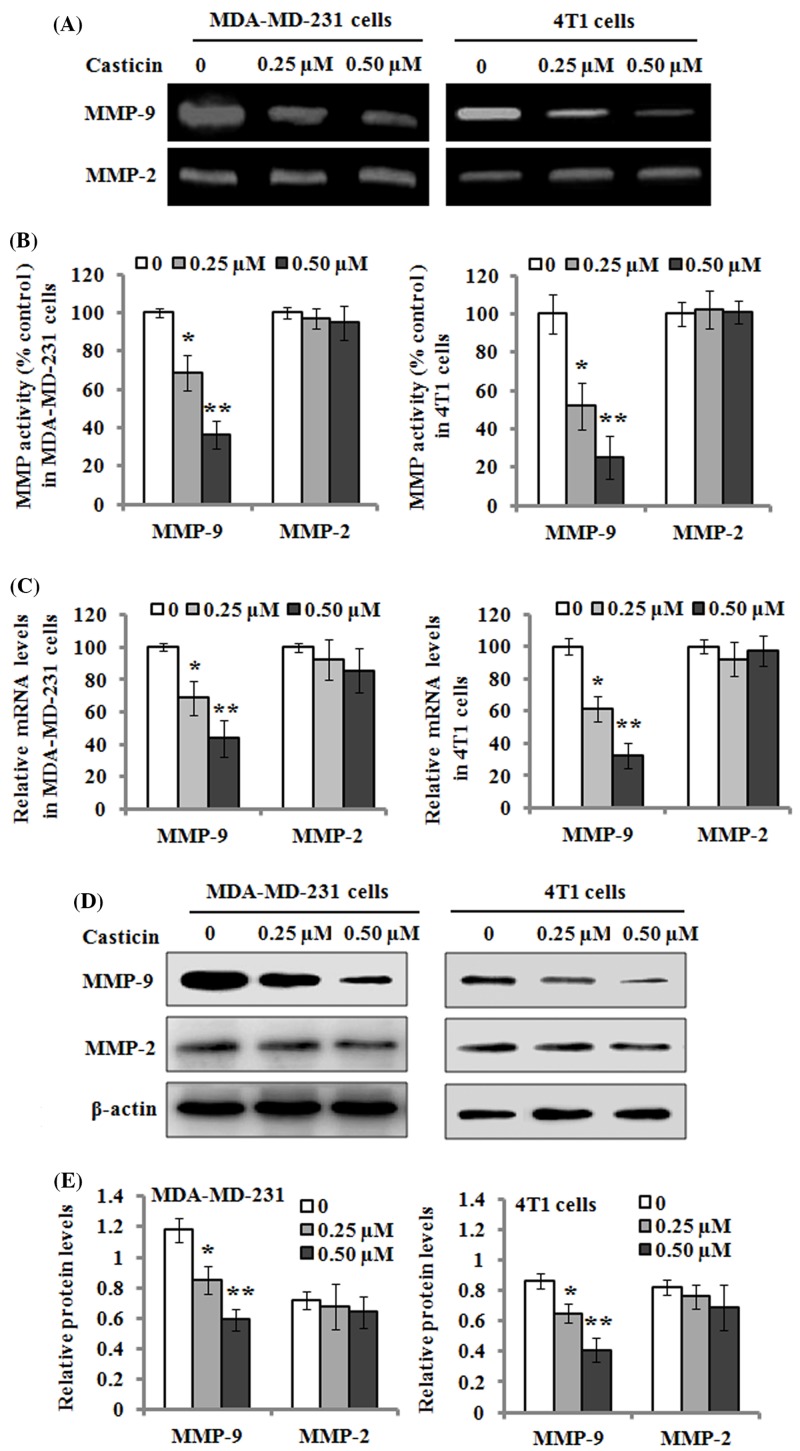
Effects of casticin on the activity and expression of MMP-2/9 (**A**) MDA-MB-231 and 4T1 cells were respectively treated with 0, 0.25, and 0.50 µM of casticin for 24 h, and the culture medium was then subjected to gelatin zymography to analyze the activity of MMP-2/9. (**B**) The activity of MMP-2/9 was separately quantitated as described in the ‘Materials and methods’ section, and normalized to that of the control. (**C**) The mRNA levels of MMP-2/9 were determined with real-time quantitative RT-PCR after the cells were incubated with 0, 0.25, and 0.50 µM of casticin for 24 h. (**D**) Western blot analysis of the protein levels of MMP-2/9 in the cells treated with 0, 0.25, and 0.50 µM of casticin for 24 h. β-actin served as an internal control for the protein level. (**E**) The relative protein levels of MMP-2/9 were quantitated against the densitometric signal of the β-actin bands. Data are expressed as the mean ± S.D. of three independent experiments. *P<0.05 and **P<0.01 compared with the control.

The effects of casticin on the activities of MMP-2/9 were analyzed by gelatin zymography. The results showed that MMP-9 activity was tremendously decreased by casticin treatment at 0.25 and 0.50 µM after 24 h incubation, whereas no significant effect on MMP-2 activity was observed in the MDA-MB-231 and 4T1 cells (Figure 3A,B). In order to investigate the regulatory roles of casticin on MMP-2/9, real-time quantitative PCR analysis was applied to evaluate the mRNA expression of MMP-2/9. The results revealed that the mRNA level of MMP-9 was significantly down-regulated by the treatment of 0.25 and 0.50 µM casticin for 24 h, but no effect was observed on MMP-2 expression (Figure 3C). Besides, Western blot results showed that the casticin-mediated changes in the mRNA levels of MMP-2/9 coincided well with their protein levels (Figure 3D,E). These findings suggest that MMP-9 activity and its expression might be regulated by casticin.

### Effects of casticin on the nuclear translocation levels of NF-κB, c-Jun, and c-Fos

**Figure 4:**
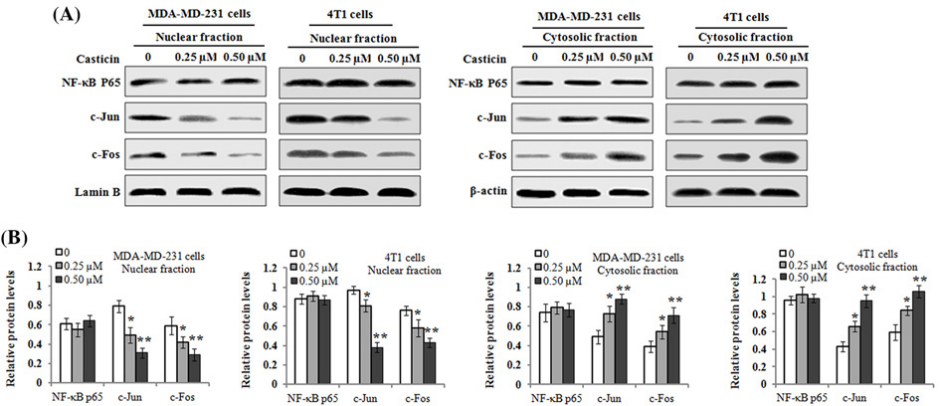
Effects of casticin on NF-κB, c-Jun, and c-Fos nuclear translocation levels MDA-MB-231 and 4T1 cells were treated with 0, 0.25, and 0.50 µM of casticin for 24 h, respectively. Nuclear and cytosolic protein extracts were subjected to Western blot analysis. (**A**) Representative results of Western blotting for NF-κB, c-Jun, and c-Fos in the nuclear fractions and cytosolic extracts, respectively. Lamin B and β-actin served as an internal control for nuclear and cytosolic fractions, respectively. (**B**) The protein levels of NF-κB, c-Jun, and c-Fos in the nuclear fractions and cytosolic extracts were respectively quantitated against the densitometric signal of Lamin B or β-actin bands. Data are expressed as the mean ± S.D. of three independent experiments. *P<0.05 and **P<0.01 compared with the control.

Previous reports have indicated that MMP-2/9 gene expression is transcriptionally regulated by various transcription factors such as NF-κB, c-Jun, and c-Fos (components of transcription factor, Activator protein-1 (AP-1)) [23,24]. Therefore, we sought to detect whether casticin perturbed the translocation of NF-κB, c-Jun, and c-Fos into the nucleus by Western blot analysis. As shown in Figure 4, the results demonstrated that the levels of c-Jun and c-Fos proteins in the nucleus in the MDA-MB-231 and 4T1 cells were markedly reduced by casticin treatment. On the other hand, an increased level of c-Jun and c-Fos proteins was found in the cytosolic extracts from the cells treated by casticin. However, there was no noticeable change in the level of NF-κB protein expression in nuclear and cytosolic fraction under the same treatment conditions. Therefore, these findings indicate that casticin treatment resulted in an inhibitory effect on the nuclear translocation of c-Jun and c-Fos in MDA-MB 231 and 4T1 cells.

### PI3K/Akt signaling involves the casticin-mediated inhibition on cell invasion

**Figure 5:**
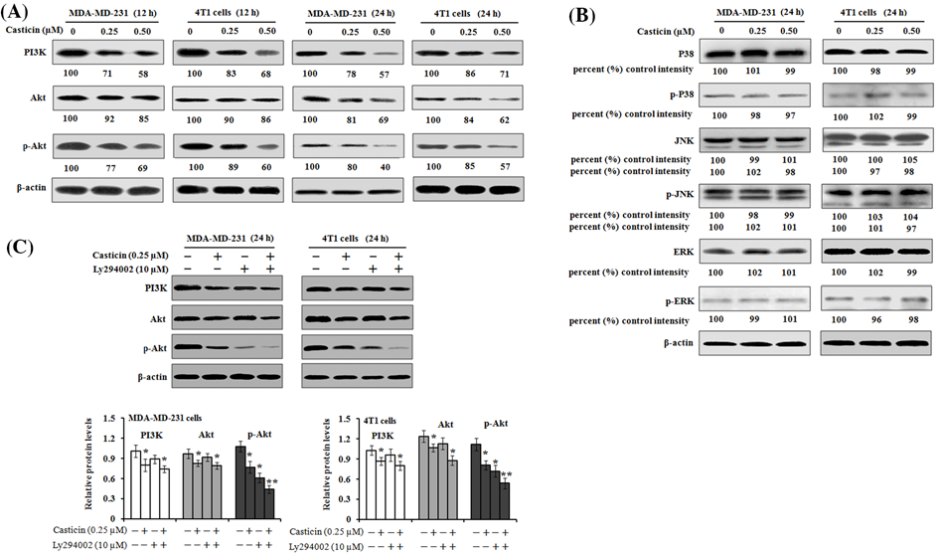
Effects of casticin on PI3K/Akt and MAPK signaling in MDA-MB-231 (**A**,**B**) MDA-MB-231 and 4T1 cells were treated with 0, 0.25, and 0.50 µM of casticin for 12 and 24 h, respectively. Cell lysates were then prepared and subjected to SDS/PAGE analysis. Representative images of Western blotting for PI3K/Akt (PI3K, Akt, and p-Akt) and MAPK (P38, p-P38, JNK, p-JNK, ERK, and p-ERK) expression. The levels of protein expression were then quantitated by densitometric analyses with that of the control at 100% as shown below the gel data. (**C**) Cells were treated with 0.25 µM of casticin for 24 h or pre-treated with 10 µM of LY294002 for 2 h prior to the addition of casticin. Protein was then harvested after treatment and the relative expression of PI3K, Akt, and p-Akt was detected by Western blotting. β-actin was used as a loading control. *P<0.05 and **P<0.01 compared with the control.

Recent studies have indicated that PI3K/Akt and MAPK signaling pathways play an important role in cancer cell migration and metastasis [25,26]. Therefore, we investigated whether PI3K/Akt and MAPK signaling pathways were involved in the casticin-mediated inhibition on cell migration and invasion. The results of Western blot analyses showed that incubation of MDA-MB-231 or 4T1 cells with casticin at 0.25 and 0.50 µM resulted in an inhibitory effect on PI3K, Akt, and p-Akt protein expression (Figure 5A), while no effects on the MAKP signaling pathway including P38, JNK, and ERK expression and their phosphorylated levels were found (Figure 5B). To further confirm the function of the PI3K/Akt pathway, a classic PI3K inhibitor, LY294002 was used. The activation of p-Akt was found to be significantly inhibited by casticin (0.25 µM for 24 h) or LY294002 (10 µM for 2 h). The combined treatment with these two reagents resulted in a further enhanced inhibition on p-Akt expression (Figure 5C). These results indicate that the PI3K/Akt signaling pathway is inhibited when the cells are treated with casticin.

**Figure 6:**
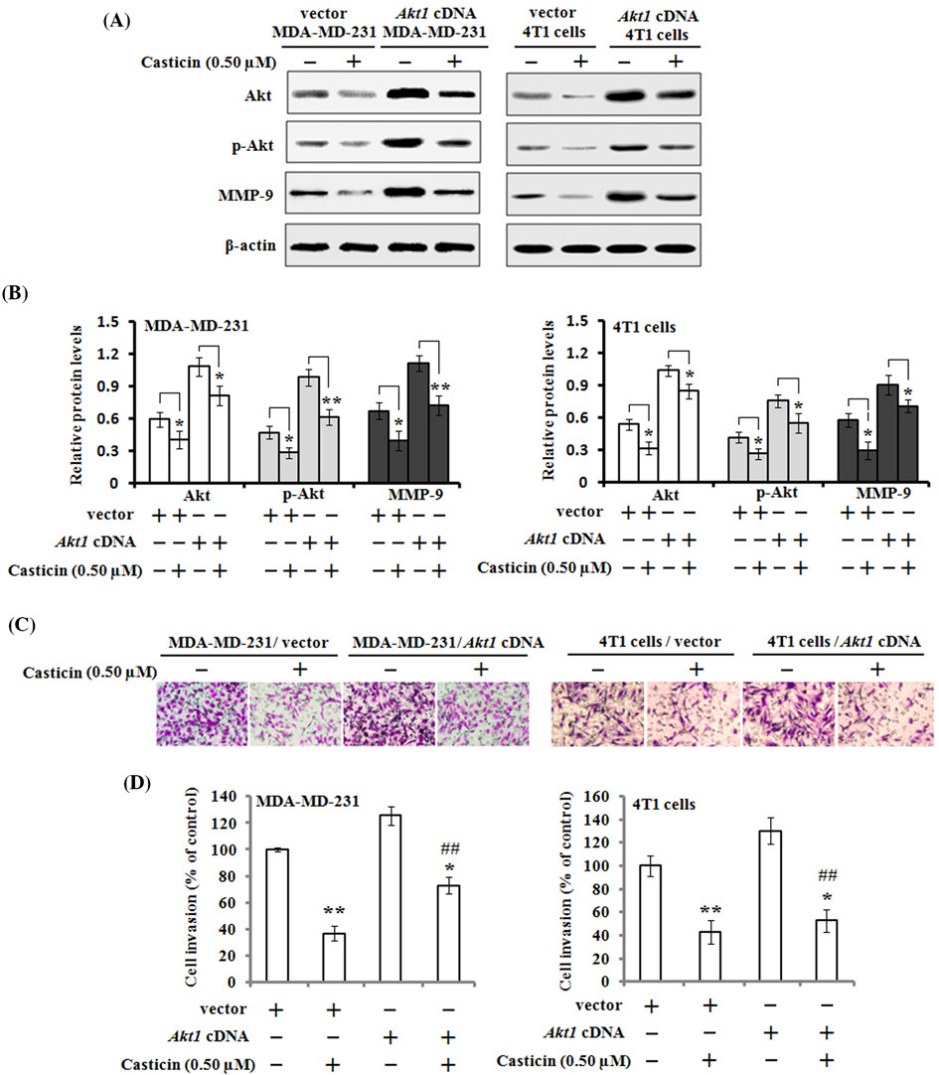
Effects of mutant Akt expression vector on casticin-mediated cell invasion and its related proteins MDA-MB-231 and 4T1 cells were respectively transfected with *Akt1 cDNA* or empty vector, and then treated with or without 0.50 µM of casticin for 24 h. (**A**,**B**) The protein expression levels of Akt, p-Akt, and MMP-9 were analyzed by Western blotting and quantitated against the densitometric signal of β-actin bands. *P<0.05 and **P<0.01. (**C**,**D**) Cell invasion was analyzed by Boyden chamber assays, and the quantitative data were presented as mean ± S.D. of three independent experiments. *P<0.05 and **P<0.01 compared with the control. ^##^*P*<0.01 compared with the untreated MDA-MB-231/*Akt1* cDNA group or 4T1/*Akt1* cDNA group.

In order to further confirm the involvement of the PI3K/Akt signaling pathway in casticin-mediated suppression of cell migration and invasion, Akt overexpression was performed in MDA-MB-231 and 4T1 cells, respectively. Western blot results demonstrated that the cells transfected with a control empty vector had decreased protein levels of Akt, p-Akt, and MMP-9 when the cells were exposed to 0.5 µM casticin for 24 h, but this inhibitory effect of casticin was reversed by Akt overexpression (Figure 6A,B). Akt overexpression in the MDA-MB-231 and 4T1 cells improved their invasive abilities, but casticin treatment still significantly attenuated their invasive abilities as analyzed by transwell Matrigel invasion assays (Figure 6C,D). These results indicate that casticin inhibits cell migration and invasion possibly by suppressing the PI3K/Akt signaling pathway.

### *In vivo* anti-metastatic effect of casticin

**Figure 7:**
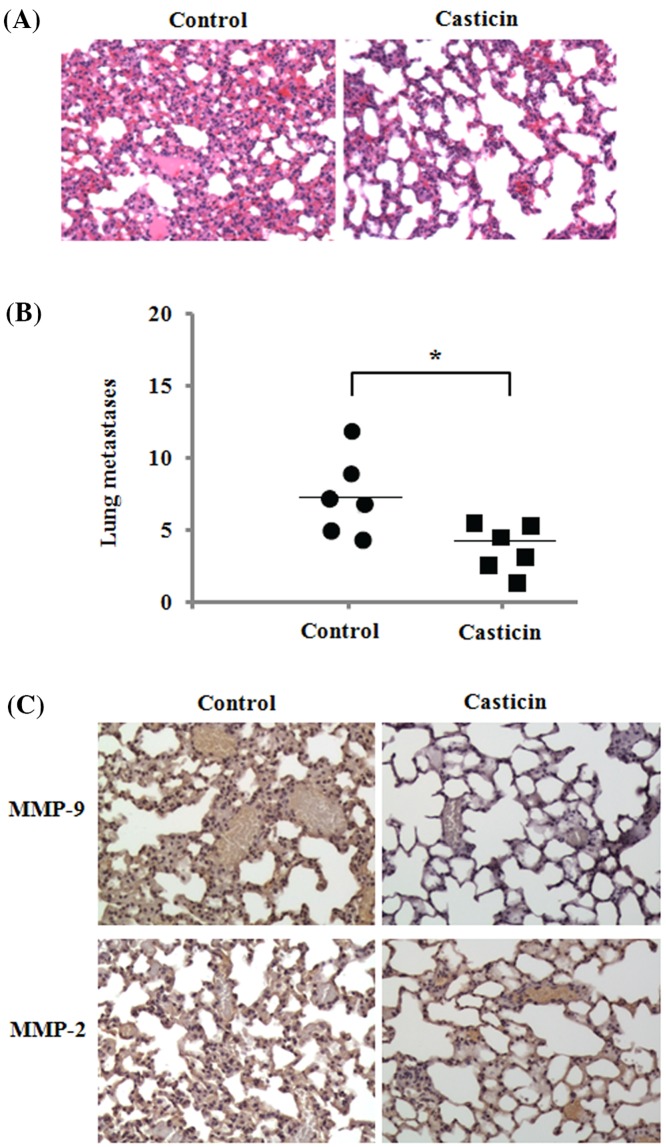
Inhibitory effect of casticin on experimental lung metastasis of 4T1 cells Mouse 4T1 breast cancer cells were implanted into the left ventricle of the heart in female Balb/c mice, and received 10 mg/kg casticin (i.p.) once every 2 days for 4 weeks. (**A**) Representative images of lung sections (H&E staining) from the control and casticin-treated mice. (**B**) Number of lung metastases detected by H&E staining of the sections. *P<0.05 compared with the control. (**C**) Representative images of the immunohistochemistry analysis of MMP-2 and MMP-9 expression in lung tissues in control and casticin-treated mice. Original magnification: 200×.

To investigate whether casticin treatment has an anti-metastatic effect, we employed an animal model of hematogenous metastatic dissemination. We implanted highly invasive 4T1 cells into the left ventricle of the heart of mice, and treated them with casticin by i.p. injection once every 2 days for 4 weeks. The results demonstrated that the mice treated by casticin showed a significant decrease in lung metastases on evaluation of H&E-stained lung sections as compared with the control mice (Figure 7A,B). Additionally, the results also demonstrated a decreased expression of MMP-9 in lung tissues of mice treated by casticin, but no obvious effect on MMP-9 expression (Figure 7C). These data suggest that the inhibition of MMP-9 expression is involved in casticin-induced inhibitory effect against breast cancer cell metastasis.

## Discussion

Breast cancer is one of the most common cancers and the major cause of mortality due to cancer amongst women worldwide [27]. Although tremendous progress in breast cancer diagnosis and therapy has been made, the 5-year survival rate and prognosis for patients with breast cancer is still not ideal [28]. Its high mortality rate is associated with cancer cell metastasis which is a complex process involving proteolytic degradation of extracellular matrix (ECM), cell migration, adhesion, and invasion [29]. Accordingly, pharmacological inhibition on cancer cell migration and invasion is a potential strategy for cancer treatment [30]. Although previous studies revealed that casticin displayed cytotoxic effects and induced apoptosis in many human cancer cell lines via different molecular mechanisms [6,31], no information about the effects of casticin on cell migration and invasion in breast cancer cells was available. In the present study, we found that casticin at 0.25 and 0.50 µM significantly inhibited cell migration and invasion in the breast cancer MDA-MB-231 and 4T1 cells, which both have a strong ability in cell migration and invasion. These inhibitory effects were not due to a lower proliferation rate of cells compared with the control cells, since we have shown that 0.25 and 0.50 µM of casticin did not significantly affect the viability and proliferation of cells (Figure 1B,C).

Cell migration and invasion is a complex process involving proteolytic degradation of ECM. It is well-known that matrix metalloproteinases (MMPs) can degrade ECM and basement membrane to facilitate migration and invasion of cancer cells [32]. Additionally, elevated levels of MMPs are functionally linked to cancer cell metastasis [33]. Therefore, MMPs play a critical role in cancer cell migration and invasion. In the present study, the activity of MMP-9 was found to be decreased by casticin in breast cancer cells, as confirmed by gelatin zymography. Besides, casticin treatment down-regulated the mRNA and protein levels of MMP-9, not MMP-2 in the cells. In the animal model, the decreased MMP-9 expression was found in lung tissues of mice administered by casticin (Figure 7C). These findings suggest that the suppressive effects of casticin on breast cancer migration and invasiveness are regulated by the down-regulation of MMP-9 activity and its expression. Similarly the decreased activity of MMP-9 was also found in the casticin-mediated inhibitory effect on the invasion of lung cancer stem-like cells [15]. MMP-2 and MMP-9 belong to gelatinase-A (72 kDa) and gelatinase-B (96 kDa), respectively, and they are both key enzymes that control the rate of cell invasion and metastasis [34]. Previous studies have indicated that MMP-2 and MMP-9 have similar properties, but their gene expression is differentially and specifically regulated by distinct regulatory elements in their promoter regions [35]. MMP-2 was found to be commonly constitutively expressed in various tissues including malignant neoplasms, rather than as part of an initial response to invasion [36]. However, MMP-9 synthesis and its secretion can be stimulated by various inflammatory cytokines and growth factors during pathological processes [37]. AP-1 and NF-κB-binding sites as inducible promoter elements were found to regulate MMP-9 gene transcription, but not MMP-2 [38]. Therefore, these differences probably accounted for casticin-mediated inhibitory effects on the activity and expression of MMP-9 in breast cancer MDA-MB-231 and 4T1 cells. Previous studies have indicated that MMP-9 gene expression can be regulated at the transcriptional level through NF-κB or AP-1 transcription factors [39,40]. It is well-known that c-Jun and c-Fos are two main components of AP-1. C-Jun and c-Fos are immediate-early genes [41]. The elevation of NF-κB or c-Jun and c-Fos nuclear translocation eventually up-regulates MMP-9 expression, which is important for cancer cell invasion. In the present study, we investigated the effects of casticin on the nuclear translocation of NF-κB, c-Jun, and c-Fos in the breast cancer cells. Our findings revealed that casticin treatment blocked the translocation of c-Jun and c-Fos into the cellular nucleus in MDA-MB-231 and 4T1 cells, as confirmed by Western blot analyses, which displayed that the levels of c-Jun and c-Fos proteins in nuclear fractions were markedly reduced, whereas their protein levels in cytosolic fractions were significantly increased after casticin treatment. However, casticin treatment showed little effect on the level of NF-κB protein in the nuclear and cytosolic extractions. These results suggest that the suppression of MMP-9 expression was probably due to casticin-mediated blockage of c-Jun and c-Fos from the cytoplasm into the nucleus in the breast cancer cells.

Previous studies have documented that the PI3K/Akt signaling pathway is involved in cancer cell migration and invasion in various kinds of cancers [42,43]. The activation of PI3K/Akt and their downstream factors, NF-κB, c-Jun, and c-Fos have been reported to increase the expression of MMPs and to proceed to promote cancer invasion [44]. Additionally, it has been indicated that MAPK families, including JNK, ERK, and P38 kinase are also involved in cell invasion and cancer metastasis [45]. MAPK families have an important role in the activation of AP-1 through c-Jun or c-Fos phosphorylation [46]. Therefore, in the present study, we investigated the effects of casticin on PI3K/Akt and MAPK signaling pathways in the MDA-MB-231 and 4T1 cells. We found that casticin treatment significantly down-regulated the protein levels of PI3K, Akt, and p-Akt in the cells, but no noticeable impact on the expression of P38, JNK, ERK, and their corresponding phosphorylation levels was observed. Consistent with our findings, Liu et al. [15] reported that casticin treatment reduced MMP-9 activity and down-regulated Akt phosphorylation in lung cancer stem-like cells. The combined treatment with casticin and a classic PI3K inhibitor, LY294002, resulted in a further down-regulation of the p-Akt level. These findings suggest that casticin inhibited cell migration and invasion possibly through inactivation of PI3K/Akt signaling in breast cancer cells, not MAPK signaling. Further investigation found that MDA-MB-231 and 4T1 cells transfected with *Akt1* cDNA demonstrated an increase in Akt and its phosphorylation and MMP-9 expression. The overexpressed Akt in the transfected cells were markedly down-regulated after casticin treatment. Additionally, the activation of Akt in these transfected cells resulted in an increase in cell invasion, but casticin treatment could significantly suppress their invasion as measured by a Boyden transwell chamber (Figure 6). Therefore, PI3K/Akt signaling undoubtedly played a crucial role in casticin-mediated inhibitory effects on cell migration and invasion in the MDA-MB-231 and 4T1 cells.

Metastasis is a complex process in which tumor cells acquire the ability to spread to other tissues through lymphatics or blood vessels, and it has been reported to be the major cause of mortality in patients with breast cancer [47]. To investigate whether casticin can affect breast cancer cell metastasis, a model of experimental lung metastasis in mice was performed. 4T1 cells are highly invasive and can metastasize to multiple sites, including the lung [48]. The results showed that the metastatic nodules in lung tissue sections evaluated by H&E staining were significantly decreased in casticin-treated mice as compared with control group mice. These results are consistent with the inhibitory effects of casticin on cell migration and invasion *in vitro*. These data suggest that casticin has a potent inhibitory effect against experimental lung metastasis. It is worth noting that we only detected the metastasis of 4T1 cells to lung tissues in this model of lung metastasis in the mice. Casticin treatment probably has the ability to inhibit 4T1 cell metastasis to other organs, such as liver, bone, spleen, and kidney, which needs to be investigated further in future research.

**Figure 8:**
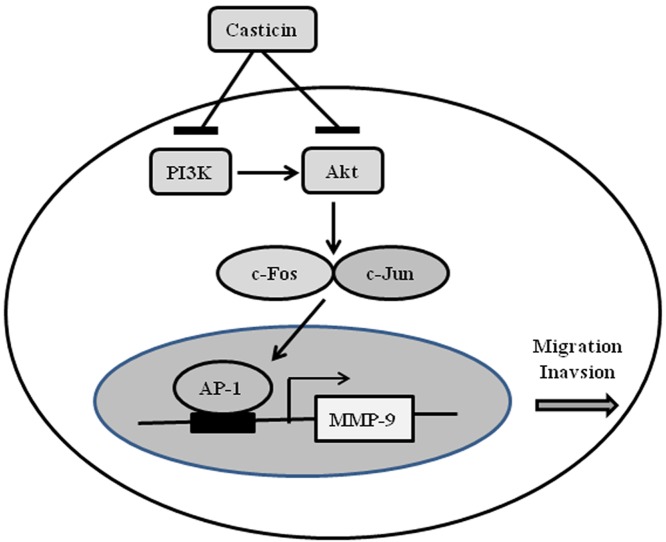
A proposed diagram for the casticin-mediated inhibitory effects on the migration and invasion of breast cancer cells

In summary, based on our findings, we proposed a schematic presentation of possible mechanisms for the suppressive effects of casticin on migration and invasion capability in breast cancer cells (Figure 8). This is the first work to reveal that casticin treatment inhibited breast cancer cell migration and invasion through down-regulation of the PI3K/Akt signaling pathway and blockage of c-Jun and c-Fos nuclear translocation, which finally resulted in a decrease in MMP-9 expression. These findings suggest that casticin is a potential anti-invasion and anti-metastasis agent for the treatment of breast cancer cells.

## Abbreviations

AP-1: activator protein-1
DMEM: Dulbeccos modified Eagles medium
ECM: extracellular matrix
ERK: extracellular signal-regulated kinase
GAPDH: glyceraldehyde-3-phosphate dehydrogenase
H&E: Hematoxylin and Eosin
i.p.: intraperitoneal
JNK: c-Jun N-terminal kinase
MAPK: mitogen-activated protein kinase
MMP: matrix metalloproteinase
NF-κB: nuclear factor-κB
PI3K: phosphatidylinositol 3-kinase
