# Expression of Concern: Casticin inhibits breast cancer cell migration and invasion by downregulation of PI3K/Akt signaling pathway

**DOI:** 10.1042/BSR-20180738_EOC

**Published:** 2021-03-31

**Keywords:** Breast cancer, Casticin, invasion, MMP-9, PI3K/Akt

The Editorial Office has been made aware of potential issues surrounding the scientific validity of this paper, hence has issued an expression of concern to notify readers whilst the Editorial Office investigates.

